# Knowledge mapping of osteoporotic fractures: a bibliometric analysis from 2014 to 2024

**DOI:** 10.1007/s11657-025-01620-6

**Published:** 2025-12-04

**Authors:** Renwei Cao, Zhongyu Wang, Shuai Lu, Minjuan Li, Xian Zhao, Hao Sun, Xieyuan Jiang, Yejun Zha

**Affiliations:** 1 Department of Orthopaedics and Traumatology, Beijing Jishuitan Hospital, Capital Medical University, Beijing, China; 2 Peking University Fourth School of Clinical Medicine, Beijing, China; 3 National Center of Orthopaedics, Beijing, China; 4 Beijing Research Institute of Traumatology and Orthopaedics, Beijing, China

**Keywords:** Osteoporosis, Fractures, Bibliometric, Visualization, Global trend

## Abstract

**Background:**

Osteoporosis and subsequent fractures not only seriously threaten the health of the aging but also escalate healthcare costs and social burdens. Undoubtedly, it is necessary to constantly optimize the diagnosis and treatment of osteoporotic fractures, and further understanding and investigation of the related studies is indispensable. However, the research on osteoporotic fractures is difficult to summarize, with complicated content and multiple disciplines involved. Consequently, this bibliometric analysis was conducted to gain a comprehensive understanding and grasp the hot topic of osteoporosis worldwide, which is beneficial to precisely guide research trends and demonstrate emerging treatments and preventive strategies.

**Methods:**

The literature published between January 2014 and October 2024 was systematically searched in the Web of Science Core Collection (WoSCC). Visualization software, including Vosviewer and CiteSpace, was used to conduct the analysis.

**Results:**

A total of 6682 publications were obtained from 2014 to 2024 for bibliometric analysis. The USA is far ahead of other countries in both the number of publications and frequency of citations, with extensive co-authorship collaborations with nearly all other countries. Cyrus Cooper is the most influential author, and the University of Sheffield is the most influential organization in the related fields. The keywords are categorized as 6 clusters, including “hip fracture,” “bone turnover,” “denosumab,” “atypical femoral fracture,” “vitamin D,” and “bone strength”. The ongoing hotspots include “teriparatide,” “hip,” “fragility fractures,” and “outcome”.

**Conclusion:**

This study comprehensively summarizes the topic trends and developments in the field of osteoporotic fractures using bibliometric analysis over the past decade. The hotspots and frontiers mainly focused on the prediction of osteoporosis and fractures, drug treatment, and clinical management. The intersection of multiple disciplines and various emerging frontiers has provided great potential for the management of osteoporotic fractures. Further investigation and more clinical translation are necessary.

## Introduction

Epidemiologic studies have revealed that people who suffered from osteoporosis is 11% of the global more than 60 years old, and the proportion is projected to reach 22% by the year 2050 [1]. Osteoporosis is a skeletal disorder characterized by decreased bone mass and deterioration of microarchitecture of bone. According to World Health Organization, approximately 8.9 million fractures worldwide each year are attributed to osteoporosis, with over 1.5 million cases occurring annually in China alone [2–4]. As the global population ages, issues related to osteoporosis and osteoporotic fractures have become increasingly prominent. Undoubtedly, it is essential to continuously optimize the diagnosis and treatment of osteoporotic fractures, making further research and deeper understanding in this field.

The term osteoporotic fracture refers to a fracture caused by small or non-traumatic force, resulting from severe reduction in bone density and strength due to primary osteoporosis [5]. Osteoporotic fractures not only affect patients’ life quality but also escalate healthcare costs and social burdens. Therefore, gaining a comprehensive understanding of osteoporosis, a global hot topic, is crucial for accurately guiding research trends and developing effective treatment and prevention strategies. Although various basic and clinical studies on osteoporotic fractures have been conducted, there remains a lack of comprehensive bibliometric summaries in this field. Hence, this article examines the hotspots related to osteoporotic fractures from a bibliometric perspective, aiming to provide further guidance for clinical work.

Bibliometrics is the process of extracting measurable data and utilizing quantitative and qualitative perspectives to quantify and visualize published literature, thereby integrating information throughout the research process and enhancing understanding of specific research areas [6, 7]. Furthermore, it demonstrates a good understanding of intrinsic connections and distribution patterns within research literature, offering a comprehensive and visual assessment of the present research status [8]. By virtue of these distinct advantages, bibliometric analysis has been widely conducted on medical research topics in orthopedic fields, including [9, 10] postmenopausal osteoporosis [11] and disease-induced osteoporosis [12, 13]. To date, however, there has been no bibliometric study focusing on osteoporotic fractures research.

As we mentioned above, osteoporotic fractures affect a large proportion of the elderly population and pose significant health hazards. However, research related to osteoporotic fractures is challenging to summarize due to its complex nature. Moreover, the interdisciplinary characteristic further complicates the synthesis of findings and the development of comprehensive treatment guidelines. Spanning fields like endocrinology, geriatrics, orthopedics, and pharmacology. Thus, it is necessary to scheme the systemic bibliometric analysis on osteoporotic fractures to present the mainstream of this research field. The main aim of this study is to investigate the trends in the field of osteoporotic fractures and guide researchers by conducting bibliometric analyses and using visualizing tools, including VosViewer and CiteSpace, so as to make it accessible to comprehensively understand the research background and development in this field.

## Methods

### Literature source and search

The comprehensive search of literature was conducted from January 2014 to October 2024 in the Web of Science Core Collection (WoSCC). Relevant studies were searched using the following formula: (TS = Osteoporosis) AND (TS = Fracture) NOT (TS = Spine) NOT (TS = body OR cells OR animal* OR mice OR rat* OR rabbit* OR dog*).

### Inclusion and exclusion criteria

We conducted a search for related publications based on the following criteria: only articles and reviews would be included, and any non-English literature would be excluded from our study.

### Data analysis

VOSviewer (version 1.6.19, USA) and CiteSpace (version 6.2, China) were used for visualization and analysis. The records of WoSCC were exported using “Full Records and Cited References” in the content and “Pure text” in the file format, and all the data was converted into VOSviewer and CiteSpace, respectively. VOSviewer mainly conducted the following visualization and analyses: co-authorship for authors, organizations, and countries; co-occurrence for keywords; citations for authors, documents, organizations, countries, and sources; co-citation for cited references. CiteSpace was mainly applied to conduct cluster analysis based on keywords to analyze burst keywords, to analyze betweenness centrality for cited references and journals, and to visualize a time-line map based on keywords.

## Results

### Search result

**Fig. 1:**
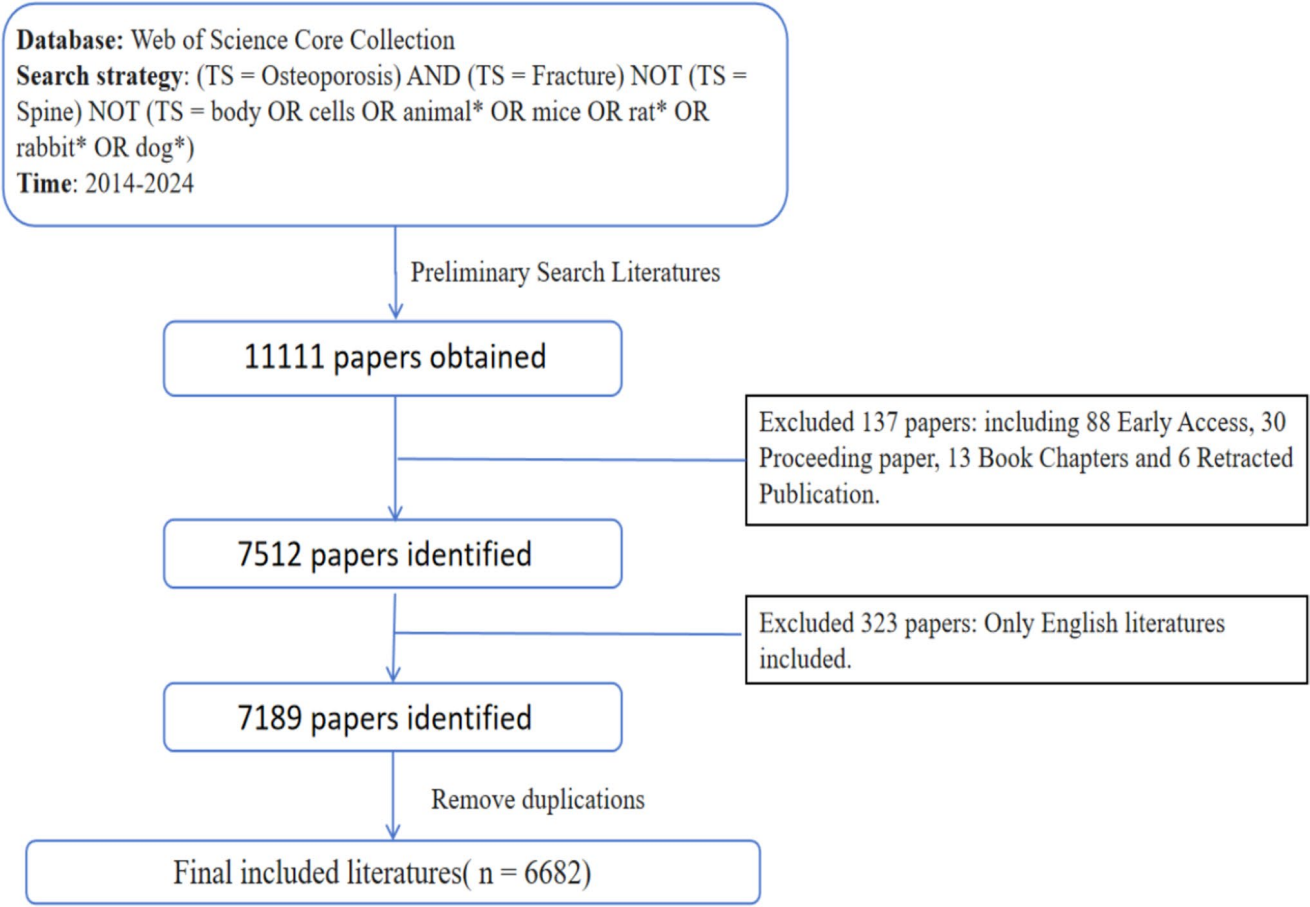
A flow diagram

The search result is shown in Fig. 1. Through search following the search strategy and imposing limitations, a total of 7189 literatures were obtained from 2014 to 2024. After recognizing and removing duplications, a total of 6682 publications were included for the final visualization and analyses.

### Number of publications

**Fig. 2:**
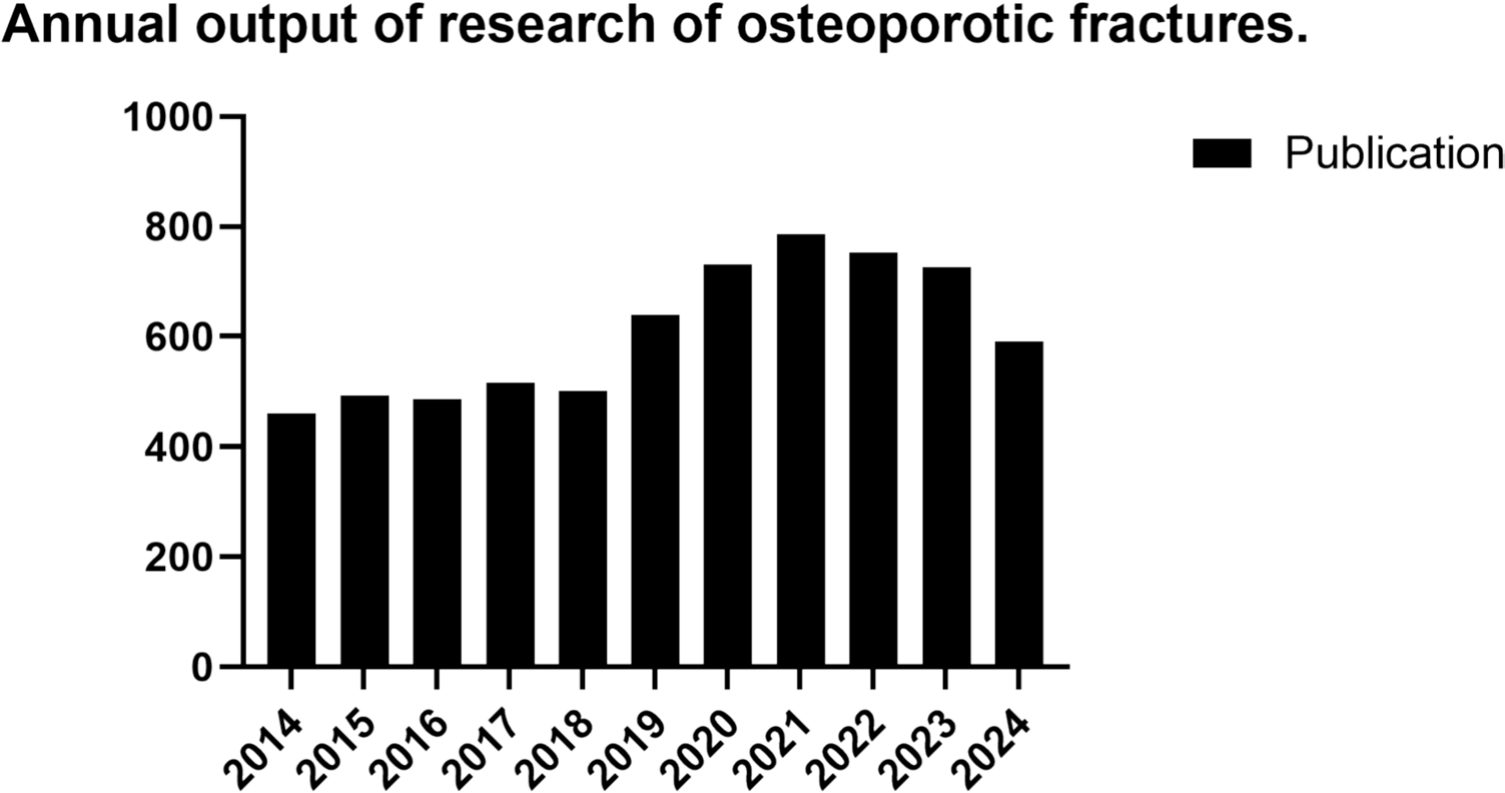
Annual output of research on osteoporotic fractures

From January 2014 to October 2024, a total of 6682 articles related to osteoporotic fracture, including 5130 articles and 1552 reviews, are finally obtained, and Fig. 2 illustrates the trend in annual publication. We found that the number of publications remained stable, averaging around 400 to 500 publications per year. This was followed by a rapid increase from 501 publications in 2018 to 786 publications in 2021, after which the numbers were stabilized.

## Authors

A total of 29,903 authors were recognized to participate in studies of osteoporotic fractures, among which C. Cooper, J. A. Kanis, E. V. Mccloskey, N. C. Harvey, and E. M. Lewiecki are the top 5 authors with the largest number of publications and C. Cooper, E. M. Lewiecki, J. A. Kanis, E. V. Mccloskey, and R. Rizzoli are the top 5 authors with the most citations.

**Fig. 3:**
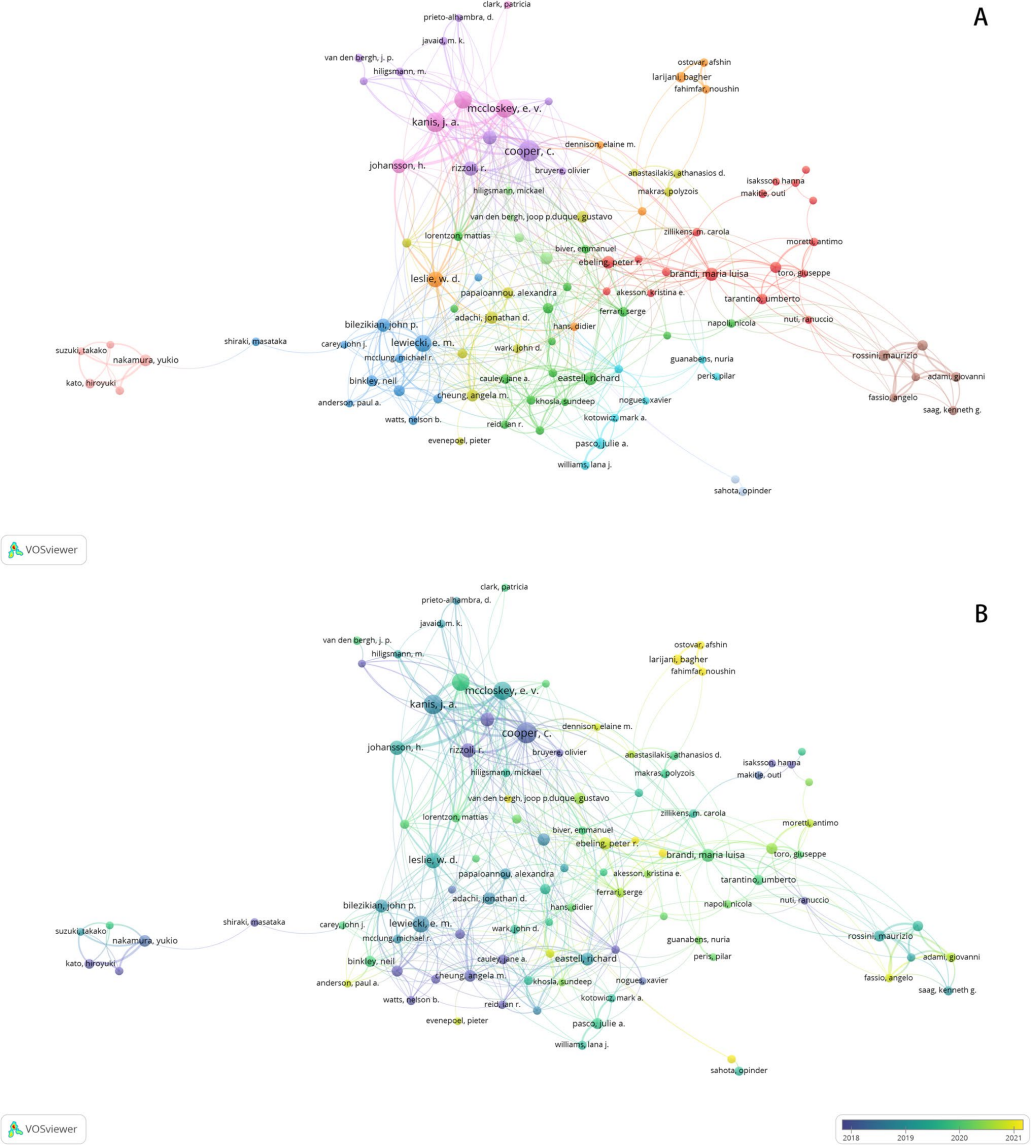
Co-authorship network analysis of authors: **A** collaborative relations in this field among different authors; **B** based on the temporal trend analysis of scholarly collaboration patterns, the color gradient reflects the recency of established cooperative relationships, with lighter hues indicating more recent years

Using a threshold of at least 10 documents, we conducted the co-authorship network analysis of authors and identified 101 nodes and 478 links. Figure 3 illustrates a dense network of collaboration among researchers, with multiple collaborative groups formed.

Besides, the overlay map of co-authorship network illustrates the meantime of authors’ research in the field of osteoporotic fractures, which is represented by colors. We found that most influential scholars, including C. Cooper, J. A. Kanis, and E. M. Lewiecki, concentrated before 2019.

### Organizations

A total of 8000 organizations are recognized to contribute to studies of osteoporotic fractures (Table 1). With no less than 100 publications, and Univ Sheffield and Univ Toronto are far ahead of other organizations with more than 130 publications. As for the citations, Univ Sheffield, Univ Southampton, Univ Oxford, and Columbia Univ rank in the top 4, with more than 5000 citations. Univ Sheffield is significantly ahead of other organizations with more than 8000 citations.

**Table 1:** The top 10 institutions ranked by citation count and publication output

Rank	Organizations	Count	Organizations	Citations
1	Univ Sheffield	135	Univ Sheffield	8212
2	Univ Toronto	132	Univ Southampton	6778
3	Univ Southampton	111	Univ Oxford	5538
4	Univ Oxford	111	Columbia Univ	5506
5	Univ Melbourne	100	New Mexico Clin Res & Osteoporosis Ctr	3826
6	Mcmaster Univ	98	Australian Catholic Univ	3637
7	Harvard Med Sch	96	Univ Liege	3175
8	Columbia Univ	89	Mayo Clin	3012
9	Monash Univ	76	Brigham & Women’s Hosp	2793
10	Univ Calif San Francisco	75	Univ Calif San Francisco	2699

The co-authorship network analysis regarding institutions was conducted using a threshold of at least 20 publications, and a total of 151 nodes and 2071 links were identified. The result reveals the collaborative network between institutions formed, including the network between the Univ of Sheffield, Univ of Southampton, Univ of Oxford, and Univ Hospital Southampton NHS Foundation Trust, the network between Univ of Toronto, St. Michael’s Hospital, Mcmaster Univ, and Univ of Mcgill, the network between Harvard Medical School, Brigham and Women’s Hospital, Beth Israel Deaconess Medical Center, and Mayo Clinic, as well as the network between Univ of Melbourne, Univ of Deakin, and Univ of Monash.

### Countries

A total of 110 countries have contributed to the research on osteoporotic fractures. As shown in Table 2, USA, China, and UK rank top 3 in the number of publications with more than 600 publications. Additionally, USA is far ahead of other countries with nearly 1600 publications. As for citations, USA and the UK rank top 2 with more than 20,000 citations.

**Table 2:** The top 20 countries ranked by citation count and publication output

Rank	Countries	Count	Countries	Citations
1	USA	1597	USA	43,742
2	People’s R China	962	UK	23,130
3	UK	690	Australia	14,124
4	Italy	496	Italy	12,817
5	Canada	451	People’s R China	12,305
6	Australia	440	Canada	11,144
7	Japan	381	Switzerland	9104
8	Germany	344	Belgium	8184
9	Spain	315	Germany	7899
10	Netherlands	257	Netherlands	7441
11	France	238	Spain	6832
12	Switzerland	238	France	6556
13	South Korea	209	Denmark	5618
14	Belgium	204	Austria	5149
15	Sweden	204	Sweden	5092
16	India	199	Japan	4204
17	Denmark	186	Saudi Arabia	3385
18	Turkey	159	Brazil	3385
19	Austria	152	New Zealand	2607
20	Brazil	140	Scotland	2512

The co-authorship analysis of countries was conducted with a threshold of at least five documents, and a total of 76 items and 1893 links were obtained. Among these, the USA showed extensive co-authorship collaborations with various countries. In contrast, China’s co-authorship relationships are notably closer with East Asian and Southeast Asian countries.

**Fig. 4:**
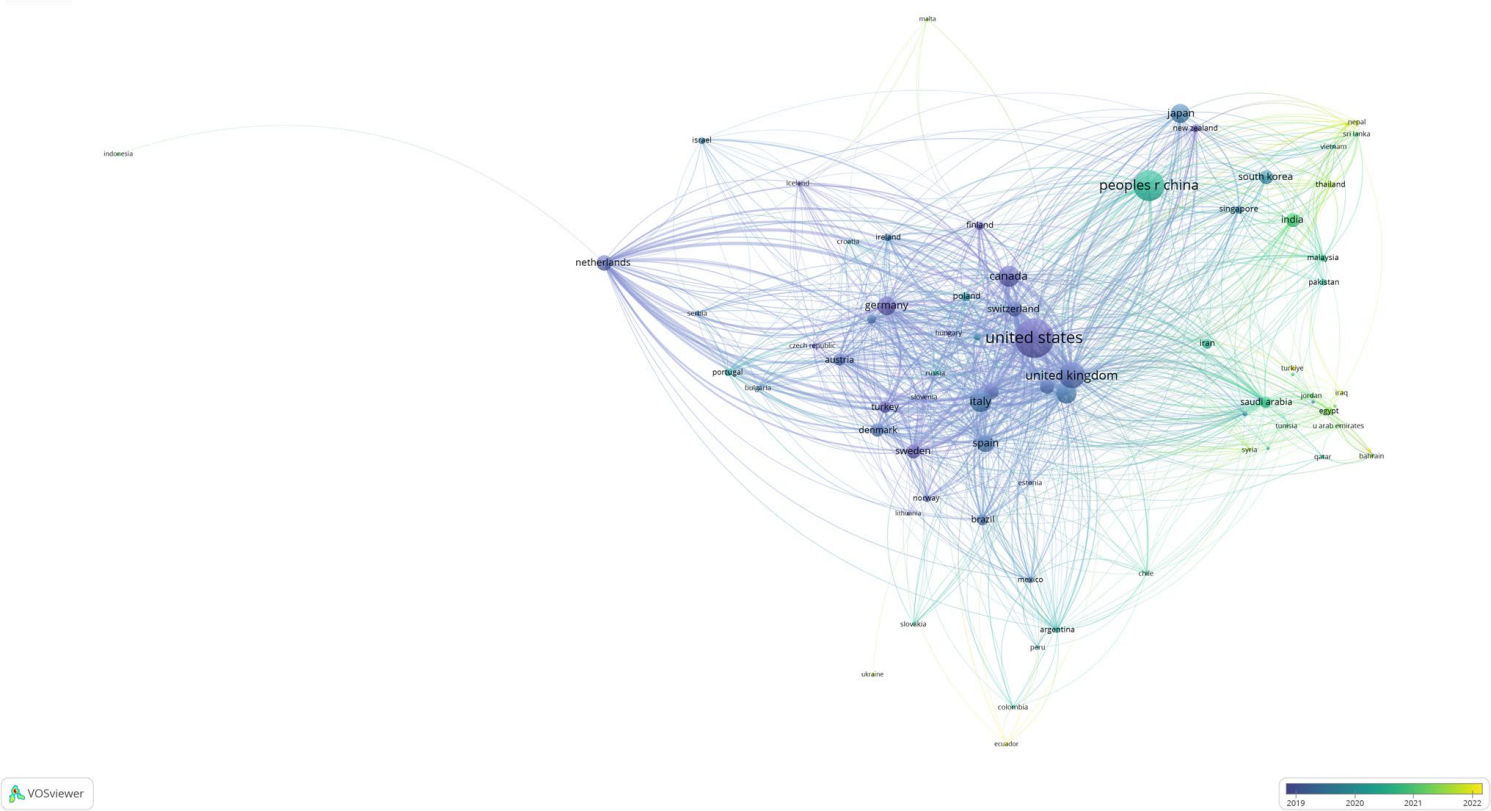
Co-authorship network analysis of countries: based on temporal trend mapping of intercountry cooperation, the color gradient reflects the timeline of partnership formation, with lighter hues denoting more recent collaborations

From Fig. 4, it is evident that developed countries such as the USA, the UK, Australia, Japan, and Germany began their research on osteoporotic fractures earlier. In contrast, China started cooperation slightly later, around 2020–2021, while countries in South Asia and West Asia, including the Philippines, Nepal, Thailand, Turkey, and Oman, initiated their cooperation research at the latest.

### Journals

A total of 1634 journals have published research in the field of osteoporotic fractures (Table 3). Counting journals’ citations, *Osteoporosis Int*, *J Bone Miner Res*, and *Arch Osteoporos* rank top 3 with more than 4000 citations. Notably, *Osteoporosis Int* is far ahead of other journals with nearly 17,000 citations. As for the number of publications, *Osteoporosis Int*, *Arch Osteoporos*, *Bone*, *J Bone Miner Res*, *Curr Osteoporos Rep*, and *Calcified Tissue Int* rank top 6 with no less than 100 publications, and *Osteoporosis Int* is also far ahead of other journals with more than 600 citations. When it comes to the impact of journals, the IFs of most journals ranked in the top 10 in both the number of publications, and citations are less than 10, and only Lancet Diabetes Endo is presented with a high IF of 44.0. The average IF of journals ranked in the top 10 in the number of publications is 7.63, while the average IF of journals ranked in the top 10 in citations is 3.07. Most journals are categorized in the Q1 or Q2 of the Journal Citation Reports (JCR), with two journals in the Q3 of JCR and one journal in the Q4 of JCR.

**Table 3:** The top 10 journals ranked by citation count and publication output along with their corresponding impact factors (IF) and JCR quartile rankings

Rank	Journals	Citations	IF/JCR	Journals	Count	IF/JCR
1	*Osteoporosis Int*	16,837	4.2/Q1	*Osteoporosis Int*	615	4.2/Q1
2	*J Bone Miner Res*	4473	5.1/Q1	*Arch Osteoporos*	273	3.1/Q1
3	*Arch Osteoporos*	4120	3.1/Q1	*Bone*	142	3.5/Q2
4	*Curr Osteoporos Rep*	3008	4.2/Q1	*J Bone Miner Res*	134	5.1/Q1
5	*Bone*	2879	3.5/Q2	*Curr Osteoporos Rep*	133	4.2/Q1
6	*J Clin Endocr Metab*	2731	5.0/Q1	*Calcified Tissue Int*	100	3.3/Q2
7	*Calcified Tissue Int*	2125	3.3/Q2	*Cureus J Med Science*	83	1.0/Q3
8	*J Clin Densitom*	1880	1.7/Q4	*J Clin Densitom*	82	1.7/Q4
9	*Injury*	1387	2.2/Q2	*J Bone Miner Metab*	76	2.4/Q3
10	*Lancet Diabetes Endo*	1344	44.0/Q1	*BMC Musculoskelet Disord*	71	2.2/Q2

### References

In the past decade, the most frequently cited references are listed in Table 4, with Cosman in *Osteoporosis International* being the most cited (Volume 25, Page 2359, https://doi.org/10.1007/s00198-014–2794-2). Among the top ten most cited articles, Cosman remains the most frequently cited, with citation counts far exceeding those of other articles. Compston and Kanis rank second and third, respectively.

**Table 4:** The top 10 most frequently cited references and the top 10 most cited documents from 2014 to 2024

Rank	References	Citations	Documents	Citations
1	Cosman, 2014, *Osteoporosis Int,* v25, p2359, https://doi.org/10.1007/s00198-014–2794-2	442	Cosman (2014b)	2112
2	Burge, 2007*, J Bone Miner Res*, v22, p465, 10.1359/jbmr.061113	373	Compston (2019)	1309
3	Cummings, 2009, *New Engl J med*, v361, p756, 10.1056/nejmoa0809493	351	Kanis (2019a)	995
4	Johnell, 2006, *Osteoporosis Int*, v17, p1726, https://doi.org/10.1007/s00198-006–0172-4	351	Birnkrant (2018)	592
5	Kanis Ja, 2008, *Osteoporosis Int*, v19, p385, https://doi.org/10.1007/s00198-007–0543-5	322	Compston (2017)	612
6	Klibanski, 2001, *Jama-J Am Med Assoc*, v285, p785	306	Morgan (2018)	475
7	Hernlund, 2013, *Arch Osteoporos*, v8, p0, https://doi.org/10.1007/s11657-013–0136-1	272	Qaseem (2017)	460
8	Shane, 2014, *J Bone Miner Res*, v29, p1, 10.1002/jbmr.1998	261	Van Dijk (2014)	440
9	Neer, 2001, *New Engl J Med*, v344, p1434, 10.1056/nejm200105103441904	258	Adler (2016)	406
10	Black, 2007, *New Engl J Med*, v356, p1809, 10.1056/nejmoa067312	242	Lips (2019)	400

**Fig. 5:**
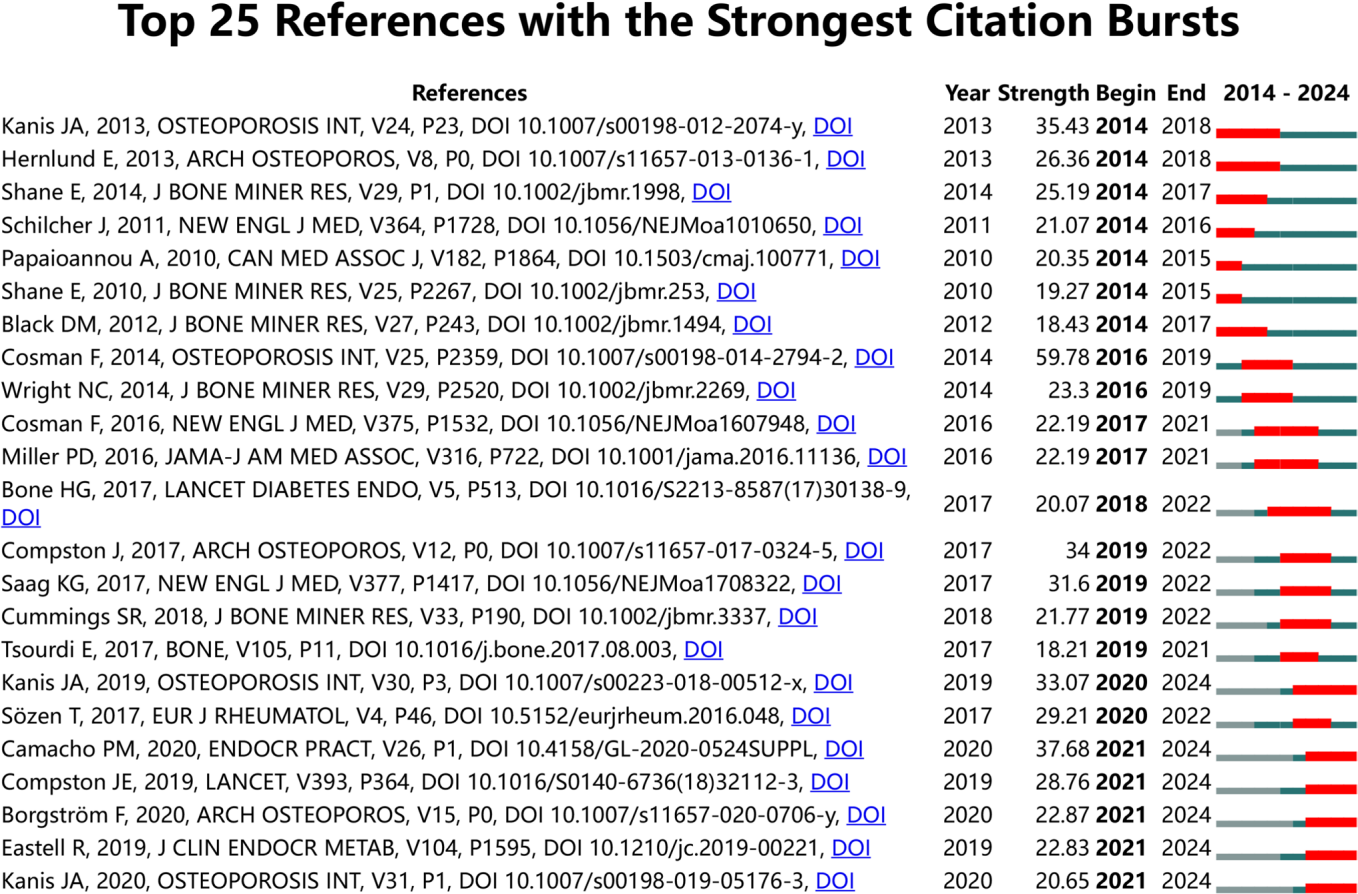
The top 25 references with the strongest citation bursts

In this study, the Top 25 references with the strongest citation bursts are identified by CiteSpace, as shown in Fig. 5. Citation bursts for references were detected starting in 2014 and continuing up until 2021. The reference with the strongest citation burst (strength = 59.78) is titled *Clinician’s Guide to Prevention and Treatment of Osteoporosis* by Cosman et al. and was published in 2014 with citation bursts between 2016 and 2019. Some references are in citation bursts until today, including Kanis, Camacho, and Compston. Overall, the strength of references ranges from 18.21 to 59.78, and the duration of citation bursts is from 1 to 4 years.

### Keyword co-occurrence

**Fig. 6:**
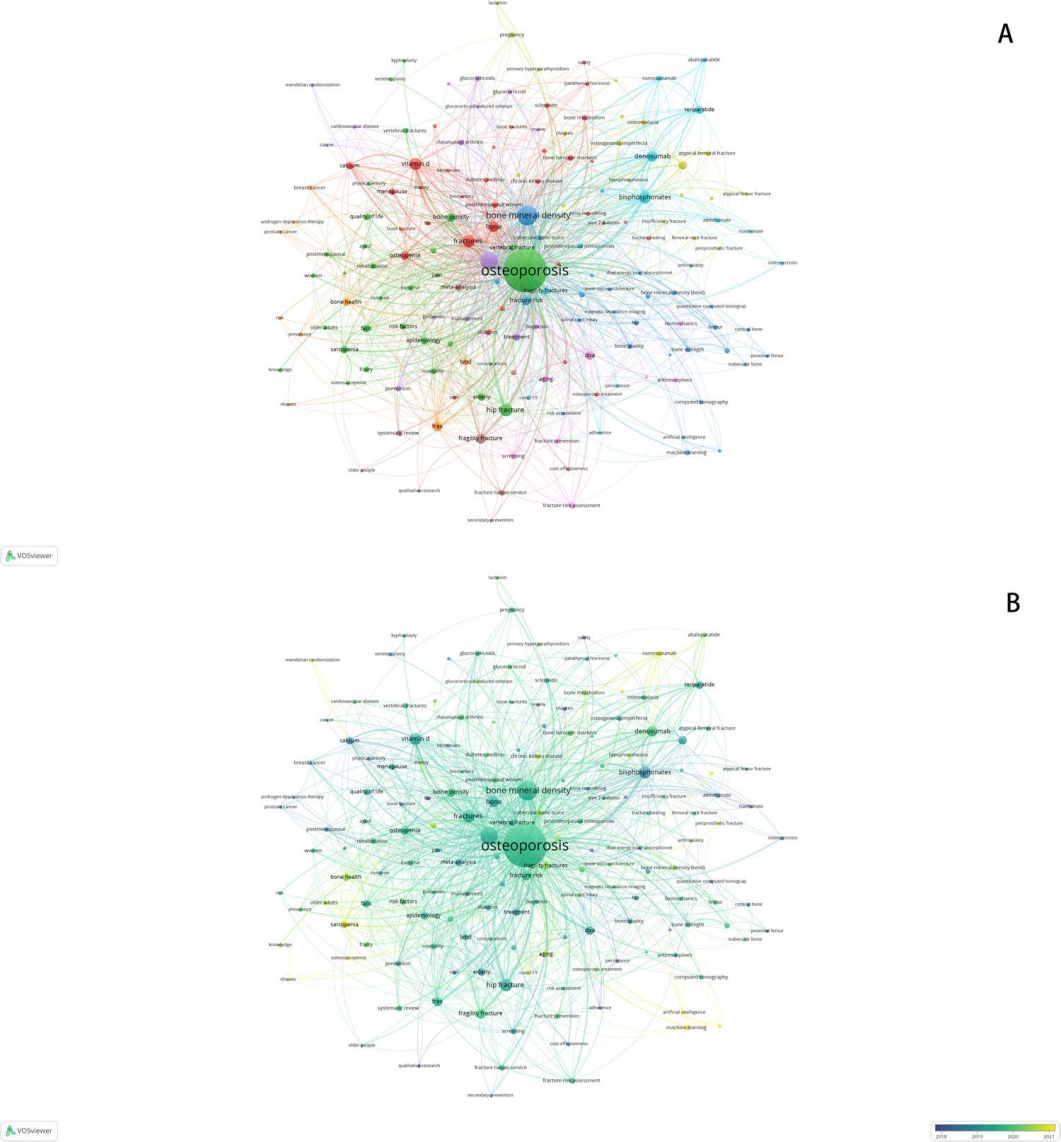
Analysis of author keywords: **A** presentation of author keywords occurrence; **B** based on the temporal trend analysis of co-occurrence of keywords, the color gradient reflects the recency of keywords, with lighter hues indicating more recent years

For keyword co-occurrence analysis, we selected Author Keywords as the unit and identified a total of 9458 keywords. With a minimum occurrence threshold of 20, a total of 166 items and 3686 links were obtained. The size of the nodes represented the frequency of occurrence, while the thickness of the lines indicated the strength of co-occurrence relationships. Figure 6 A demonstrates that the keyword “osteoporosis” has a significantly higher frequency than other keywords, followed by “bone mineral density,” “fractures,” “hip fracture,” “bisphosphonate,” and “vitamin”. Notably, “osteoporosis” shows significant co-occurrence with all other keywords “bone mineral density (BMD)” has clear co-occurrence relationships with “fracture,” “risk,” “vitamin D,” and “bisphosphonates”. Additionally, there is a notable co-occurrence between “bisphosphonates” and “denosumab”.

The overlay map of co-occurrence of keywords, shown in Fig. 6 B, illustrates the time of appearance of keywords. Most influential words, including “osteoporosis,” “bone mineral density,” “fractures,” “bisphosphonates,” “hip fracture,” and “vitamin d” are highlighted in blue or green.

**Fig. 7:**
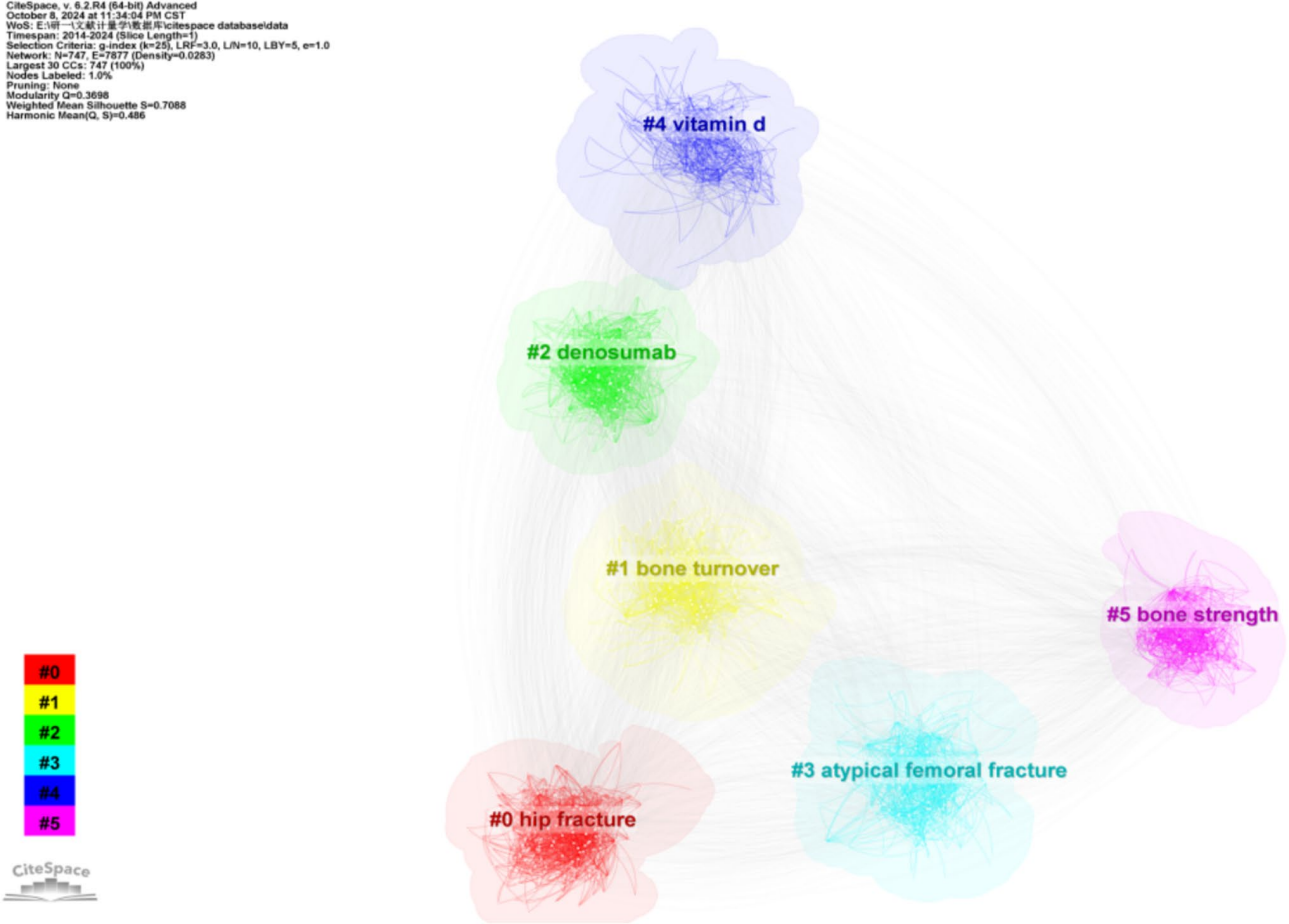
Cluster analysis of keywords, with six labels representing six keyword clusters

Using the LLR text processing method, we conducted cluster analysis based on keywords. As shown in Fig. 7, six labels representing six clusters are obtained, with each cluster label corresponding to co-occurrence network keywords. The keywords include “hip fracture,” “bone turnover,” “denosumab,” “atypical femoral fracture,” “vitamin D,” and “bone strength,” numbered 0 to 5. A higher number indicates that the cluster contains fewer keywords, while a lower number indicates a larger set of keywords. Specifically, cluster #0, which includes “hip fracture,” contains the most keywords, while cluster #5, “bone strength,” contains the fewest.

The analysis indicates that *Q* = 0.3698, which is greater than 0.3, suggesting a significant clustering effect. Additionally, *S* = 0.7088, exceeding 0.7, indicates a high level of reliability in the clustering results.

**Fig. 8:**
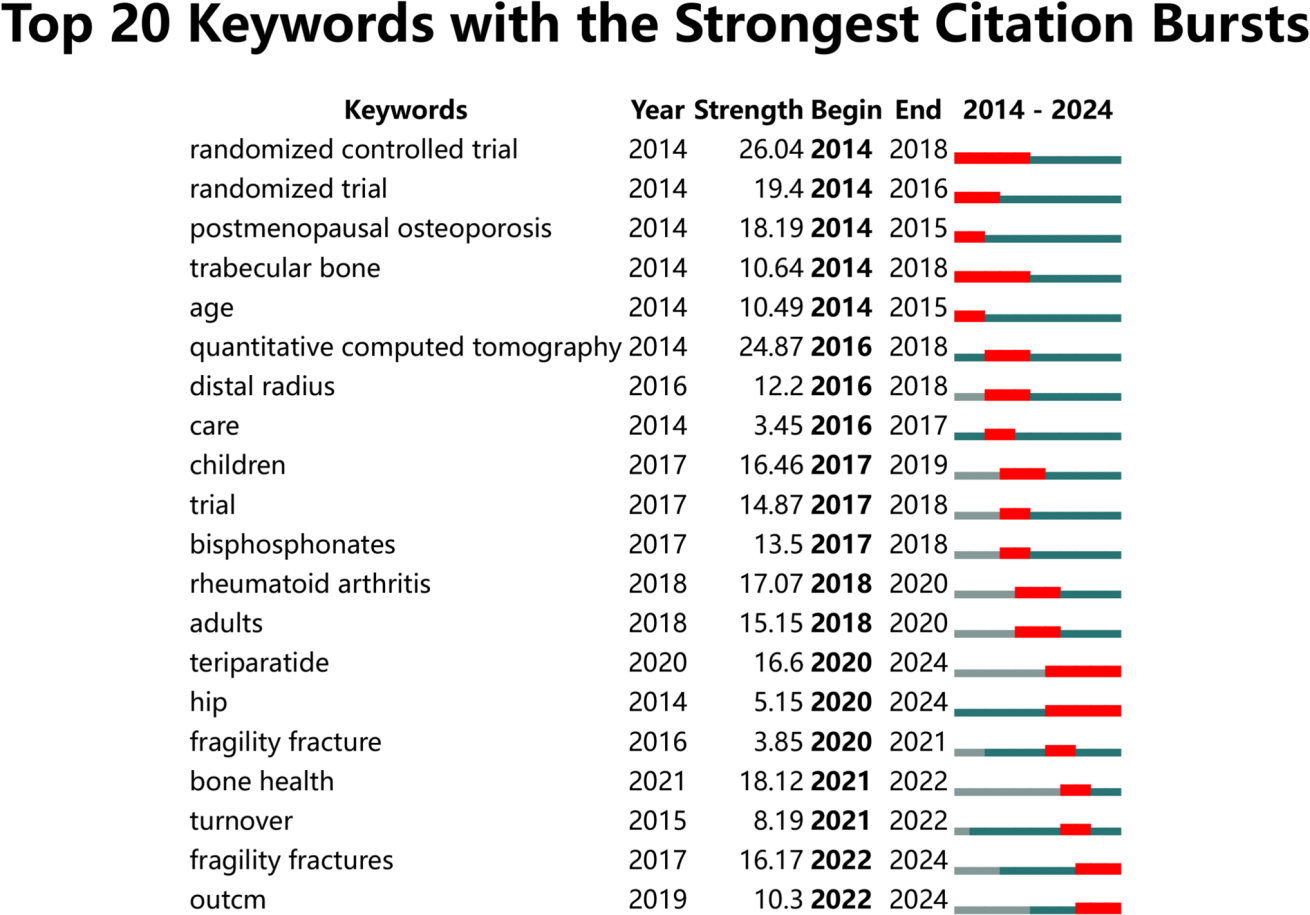
The top 20 keywords with the strongest citation bursts

Figure 8 displays the top 20 keywords related to osteoporotic fractures with the strongest mutation indices. Notably, “randomized controlled trial” has the highest mutation index, while “care” has the lowest mutation index.

### Time-line map

**Fig. 9:**
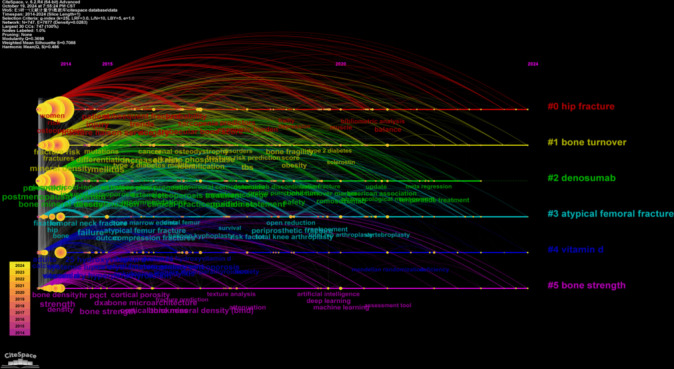
The timeline map based on six keyword clusters

The six clusters of highly relevant keywords have all emerged as early as 2014, and these key terms have maintained their relevance over the past decade. In the subsequent 10 years, each cluster has continuously introduced trending keywords, indicating the emergence of new fields or directions within these clusters. We first see that for the #0 hip fracture cluster, other prominent keywords include “fragility fracture,” “denosumab,” “fracture liaison service,” and “falls.” This cluster primarily focuses on the diagnosis and prevention of hip fractures, with most trending terms appearing no later than 2014. Notably, “fracture liaison service” began to surface in 2015. The #1 bone turnover cluster features other significant keywords such as “chronic kidney,” “diabetes mellitus,” and “bone turnover markers.” This cluster focuses on biochemical processes related to bone turnover, formation, and resorption, with all trending terms having emerged no later than 2014. In the #2 denosumab cluster, other high-impact keywords include “teriparatide,” “bisphosphonates,” “zoledronic acid,” and “romosozumab.” This cluster centers around the treatment of osteoporosis with medications, and “romosozumab” emerged in 2020, likely linked to advancements in anti-sclerostin antibody research. No new keywords have appeared in this cluster for 2024. Lastly, the #5 bone strength cluster includes other important keywords like “finite element analysis,” “trabecular bone,” “cortical bone,” and “quantitative computed tomography.” This cluster focuses on bone structure and screening of fragility fracture, with all keywords appearing no later than 2014. Notably, starting in 2020, fields such as artificial intelligence and machine learning began to emerge within this cluster. Overall, the analysis reveals a dynamic landscape in each cluster, highlighting ongoing developments and the introduction of new research areas over the past decade. The #3 atypical femoral fracture and #4 vitamin have been illustrated in detail in Fig. 9.

## Discussions

### General information

In this study, we performed a comprehensive bibliometric analysis to summarize global research trends in the field of osteoporotic fractures over the past 10 years. A total of 6682 publications were collected, published across 1643 journals contributed by 29,905 authors from 8000 organizations across 110 countries or regions. Using visualization tools such as Vosviewer and CiteSpace, we mainly conducted the co-authorship, co-occurrence keywords, co-citation cluster, burst keywords, and dual-map overlay to demonstrate the collaboration network, potential research trends, and emerging research hotspots. This systematic bibliometric analysis and mapping not only revealed the evolving intellectual structure and transnational cooperation patterns within the field but also highlights growing emphasis on areas such as prevention strategies, treatment optimization, and health-economic outcomes. These insights are critical for guiding future research directions, fostering interdisciplinary collaboration, and ultimately addressing the increasing global burden of osteoporotic fractures in aging populations.

### Analysis of results

A total of 110 countries are recognized to contribute to the research of osteoporotic fractures, and it can be seen that countries around the world have participated in the research, which is likely facilitated by international initiatives and organizations. It is obvious that the USA leads significantly in both publication volume and citation frequency, indicating its dominant position in driving scientific output and impact. Most of the top 20 countries in both publications (80%) and citations (85%) are developed countries, which may indicate that there is a striking concentration of research productivity among developed nations, suggesting a strong correlation between national economic capacity and scientific investment in osteoporotic fractures.

As for the collaboration network, the USA and the UK show extensive co-authorship collaborations with almost all countries, which may be partly explained by some international studies [14–18]. This is in line with the cooperation among countries around the world mentioned in the previous text. Moreover, this prominent positioning also underscores their role as central research hubs and potential global leaders in osteoporotic fracture research. China exhibits relatively weaker collaboration with countries in West Asia and Eastern and Southern Europe, which may be explained partly by the remote geographical position and geopolitical factors.

At the organizational level, the University of Sheffield stands out with both the most publications and citations, which indicates its prominent position in osteoporotic fractures. Besides, the University of Southampton and the University of Oxford ranked in the top 5 in both publications and citations. The leading role of these universities strengthens them as crucial sources of influential literature and suggests that their research output is essential for tracking the evolution of osteoporotic fractures. An analysis of collaboration sources indicates that most partnerships arise from collaborations between universities and between academic and clinical institutions, while involvement from businesses, industry, government agencies, or non-academic sectors is relatively scarce. This suggests that there are still plenty of significant opportunities for future translation of research findings, which could be facilitated by policies encouraging tripartite collaboration between academia, clinical institutions, and industry. Institutions or the government might be the main entities that can actively participate in the future. It is found that the collaboration network of organizations is formed consistently with the geographic position and national origins. For instance, distinct clusters emerging within the UK (Sheffield, Oxford, and Hospital Southampton NHS Foundation Trust), Canada (Toronto, McMaster, and McGill), the USA (Harvard Medical School, Mayo Clinic). Therefore, it reminds us that osteoporotic fractures may need more concerted efforts to foster international and intercontinental research and may necessitate integration of diverse populations and healthcare contexts. At the same time, it also urges us to enhance cooperation in different areas and countries.

## Journals and references

It’s obvious that *Osteoporosis International* has the highest publication number and citation frequency, significantly surpassing other journals, which underscores its leading role in osteoporotic fracture research. However, the IFs of most journals in the field are generally not high, with the average IF ranked top 10 in both publication and citation below 10. The relatively modest IFs of leading journals in this field likely reflect its strongly applied and clinical nature. Interestingly, the references ranked top 10 in citations are all categorized as clinical guides, clinical trials, or epidemiology analysis, with most attention focusing on clinical practice. Current research efforts appear to be largely focused on improving clinical applications and therapeutic strategies, validating existing treatments across diverse populations. This situation may highlight that future innovations may need to broaden horizons on integrating novel technologies, such as AI, genomics, and big data algorithms, into this established clinical framework. As the reference with the strongest citation bursts in the past decade, *Clinician’s Guide to Prevention and Treatment of Osteoporosis* by Cosman et al. in 2014 is undoubtedly of great importance in normalized prevention, risk assessment, diagnosis, and treatment of osteoporosis [19]. This not only demonstrates that it became an indispensable reference point for virtually all subsequent clinical research, establishing a new standard for best practices in osteoporotic fracture care, but also indicates its timing likely coincided with a critical mass of new evidence requiring synthesis and standardization.

### Hotspots and frontiers

Investigating the burst keywords and clustering of keywords can help grasp the hotspot in osteoporotic fractures and predict future research directions. As represented in the burst keywords, we found that the related emphasis has gradually progressed from previous clinical trial, trabecular bone, and quantitative computed tomography (QCT) to studies related to pharmacological therapies, hip fracture management, and outcomes and application of emerging technologies, indicating the deepening exploration of bone transition pathway and increasing attention on clinical prognosis. The following discussion interprets these data-driven themes within the broader context of the evolution in osteoporotic fractures.

Cluster #2 Pharmacological Therapies and strong citation bursts for keywords, such as teriparatide, romosozumab, and pharmacological therapies, highlight the evolution of pharmacological interventions as a central research frontier. Our bibliometric analysis reveals a clear translational pathway: from classic anti-resorptive agents to bone-forming drugs (teriparatide), and further to monoclonal antibodies with dual mechanisms of action (romosozumab). This progression not only reflects deepened mechanistic understanding of bone metabolism but also underscores a strategic shift from symptomatic treatment toward targeted precision medicine. As a recommended therapy for osteoporosis, anti-osteoporosis drugs, teriparatide, can significantly increase bone mineral density (BMD), improve strength and relieve clinical symptoms, and reduce the risk of re-fracture [19, 20]. In the past decade, research has further explored the clinical applications of teriparatide. It is reported that teriparatide can be used to treat femoral and vertebral fractures [21], non-union of fractures [22], and osteonecrosis of the jaw [23], achieving favorable clinical outcomes. Moreover, combination therapy involving teriparatide and denosumab has been shown to significantly enhance BMD [24], and sequential therapy has demonstrated better prognoses [25]. Another significant advancement is the development of romosozumab, a humanized monoclonal antibody targeting sclerostin. Several phase III clinical trials in the past decade [26], including FRAME, ARCH, and STRUCTURE [26–28], which have proven significant efficacy in postmenopausal osteoporosis and male osteoporosis [29–31]. This trend highlights a shift in research focus from traditional hormonal and chemical drugs toward precision therapeutics targeting specific molecular pathways. Furthermore, it reflects an advancement in pharmacological therapies—from merely increasing BMD to comprehensively improving bone quality, microarchitecture, and reducing fracture risk. The bibliometric data capture a field in transition: future research on osteoporosis medications may focus on the treatment of severe osteoporosis and sequential treatments with different drugs [32].

The high frequency and burst strength of keywords like “hip fracture” and “mortality,” along with their central position in Cluster #1, Hip fracture and Clinical management. Bibliometric analysis shows that research efforts have increasingly centered on risk prediction, epidemiological trends, economic burden, and improved clinical management. Hip fracture is a common clinical complication of osteoporosis and is often associated with poor prognoses and adverse outcomes [33, 34]. Studies have shown that hip fractures significantly impact the long-term functional recovery of elderly patients [33] and are associated with increased mortality rates [34], particularly short-term mortality and all-cause mortality. Epidemiological data indicate a rising global incidence of hip fractures [35], with distinct patterns observed between Eastern and Western populations, potentially related to urbanization rates and demographic structures [36–40]. The burst strength of keywords, such as diabetes, proton pump inhibitors, frailty, smoking, and aging, related to risk factors and post-fracture management underscores the critical importance of identifying individualized risk factors and implementing localized, specialized management strategies for hip fractures [4, 41–43]. In Southern Europe, routine CT screening for early osteoporosis detection and the implementation of the Fracture Liaison Service (FLS) model, as evidenced by concentrated keyword clusters [44, 45]. The established cost-effectiveness of the FLS model, as supported by the International Osteoporosis Foundation’s best practice framework [46], which addresses local clinical priorities and provides a translatable evidence-based model [47]. Future research may focus on clinical management for patients at high risk of fractures and those with specific fracture-related risk factors.

As Fig. 9 illustrates, hotspots have not been prominent in the past decade, and there are no very influential special clusters. In addition to the clusters, we summarized in the previous text, there are also some emerging hotspots and frontiers regarding osteoporotic fractures. Although their frequency and intensity do not form clusters, they need to be noticed and taken seriously, as clearly identified by our bibliometric analysis. The strong citation bursts and high centrality of keywords such as “machine learning,” “deep learning,” and “Mendelian randomization” mark them as defining themes of contemporary and future research directions of osteoporotic fracture [48, 49]. These studies can be broadly categorized into two types: one focuses on predicting indicators of osteoporosis (BMD, fall, and fracture risk), using clinical data and various algorithms, such as artificial neural networks (ANN), support vector machines (SVM), random forests, decision trees (DT), and deep convolutional neural networks (CNNs) [50–54], while the other involves automated segmentation tools for images of patients with osteoporosis or those at risk of developing osteoporosis [55–57]. Among these, ANN and SVM models have demonstrated superior predictive performance [50, 52, 58], and the prediction model based on ML has partly proven better performance of screening than traditional screening tools [59]. Image segmentation can be derived from multiple sources with deep learning models predominantly used for image processing and segmentation [60, 61]. The utilization of these processing models is quite complex, with a lack of evidence-based standardization [62], and there is ongoing exploration of more specialized models [63, 64], indicating a need for further research. The keywords like “standardization” and “validation” alongside these technical terms are a crucial insight from bibliometric analysis. It highlights that the major acknowledged challenge is no longer model development but the translation of these complex algorithms into standardized, reliable, and clinically actionable tools—a key frontier for future osteoporotic fractures research. Mendelian randomization is a genetic epidemiological method that utilizes genetic factors to unbiasedly clarify the associations and causal relationships between modifiable exposures and osteoporosis, providing reliable directions for the prevention of osteoporotic fractures [65]. The research focus, mapped by keyword co-occurrence, has successfully established causal links for factors like serum estradiol, BMI, and lipid levels with BMD [65–67]. However, mental health factors such as depression and bipolar disorder [24], insomnia [23], hereditary low serum 25(OH)D [68, 69], and ankylosing spondylitis (AS) [70] do not show clear causal relationships with BMD. Interestingly, hyperuricemia [71] and hypertension [72] may have a protective effect against osteoporosis. In contrast, the genetic causal relationships between rheumatoid arthritis [73, 74], inflammatory bowel disease [75, 76], and BMD remain contentious. Further research regarding detailed biological mechanisms and more clinical translation is expected and necessary.

Our bibliometric analysis reveals that research in osteoporotic fractures is structured around solid thematic clusters alongside emerging, non-clustered frontiers, such as machine learning and Mendelian randomization (Fig. 9). In summary, they highlight a field moving rapidly toward precision medicine and standardized intervention, offering a clear map for future research focused on integrating novel technologies and translating mechanistic insights into clinical practice.

### Limitations

Undoubtedly, there are certain limitations in this study. Firstly, we only searched in the Web of Science Core Collection (WoSCC) for literature, and the data from other databases were lacking, which may result in selection bias. According to our search criteria, the PubMed database, Embase database, and Cochrane database contain 7144, 6419, and 445 documents, respectively. However, the total number of included documents is 7189 from the WOS database. The main reason is that not all the data exported from the databases, for instance, PubMed, support all the analysis functions of CiteSpace and VOSviewer. Besides, studies in the field of osteoporotic fractures have been emerging since the 1960 s, and many influential keywords have appeared before 2014, while only literature published in the past decade was obtained due to an excessive number of publications. Therefore, the overall research trend in the related fields is difficult to visualize. In addition, the language type is limited only to English, which may result in some publications in other languages being missed. Consequently, we will continue to focus on the field of osteoporotic fractures, update literature in other languages, and pay attention to influential publications before 2014.

## Conclusions

Through knowledge mapping and bibliometric analysis, we systematically reviewed research in the field of osteoporotic fractures in the past decade. The number of annual publications remained a stable quantity in the first 5 years and increased steadily over the last 5 years. The USA is far ahead of other countries in both the number of publications and frequency of citations, with extensive co-authorship collaborations with nearly all other countries. The keywords are categorized as 6 clusters, including “hip fracture,” “bone turnover,” “denosumab,” “atypical femoral fracture,” “vitamin D,” and “bone strength,” respectively numbered 0 to 5, which represent different research directions. The ongoing hotspots include teriparatide, hip, fragility fractures, and outcome, which mainly focus on drug treatment of osteoporosis and prediction of hip fractures and adverse outcomes. Some newly emerging study directions, including sarcopenia, machine learning, Mendelian randomization, and romosozumab, have facilitated in-depth exploration of risk prediction, identification of genetic risk factors, and development of novel drugs.

This bibliometric study not only presents an overview of historical and current research but also highlights the increasing interdisciplinary integration and emerging hotspots. The research results provide valuable guidance for researchers, clinicians, and policymakers regarding the research directions for osteoporotic fractures. The intersection of multiple disciplines and various emerging frontiers has provided great potential for the management of osteoporotic fractures. Further investigation and more clinical translation are necessary.

## Data Availability

The data used in this study were obtained from the publicly accessible Web of Science Core Collection (WoSCC) database, provided by Clarivate Analytics. Access to the WoSCC data requires institutional subscription or individual purchase through Clarivate's official platform (https://clarivate.com/webofscience/). Researchers can query and download data directly from the database using standard search filters and export functions. For transparency, the specific search queries and filtering criteria applied in this study are detailed in the Methods section of the manuscript.
